# Testing the validity of value‐added measures of educational progress with genetic data

**DOI:** 10.1002/berj.3466

**Published:** 2018-09-09

**Authors:** Tim T. Morris, Neil M. Davies, Danny Dorling, Rebecca C. Richmond, George Davey Smith

**Affiliations:** ^1^ University of Bristol UK; ^2^ University of Oxford UK

**Keywords:** ALSPAC, attainment, education, heritability, value added

## Abstract

Value‐added measures of educational progress have been used by education researchers and policy‐makers to assess the performance of teachers and schools, contributing to performance‐related pay and position in school league tables. They are designed to control for all underlying differences between pupils and should therefore provide unbiased measures of school and teacher influence on pupil progress, however, their effectiveness has been questioned. We exploit genetic data from a UK birth cohort to investigate how successfully value‐added measures control for genetic differences between pupils. We use raw value‐added, contextual value‐added (which additionally controls for background characteristics) and teacher‐reported value‐added measures built from data at ages 11, 14 and 16. Sample sizes for analyses range from 4,600 to 6,518. Our findings demonstrate that genetic differences between pupils explain little variation in raw value‐added measures but explain up to 20% of the variation in contextual value‐added measures (95% CI = 6.06% to 35.71%). Value‐added measures built from teacher‐rated ability have a greater proportion of variance explained by genetic differences between pupils, with 36.3% of their cross‐sectional variation being statistically accounted for by genetics (95% CI = 22.8% to 49.8%). By contrast, a far greater proportion of variance is explained by genetic differences for raw test scores at each age of at least 47.3% (95% CI: 35.9 to 58.7). These findings provide evidence that value‐added measures of educational progress can be influenced by genetic differences between pupils, and therefore may provide a biased measure of school and teacher performance. We include a glossary of genetic terms for educational researchers interested in the use of genetic data in educational research.

## Introduction

Value‐added (VA) measures are frequently used by education researchers and policy‐makers to assess the performance of teachers and schools, and therefore impact upon performance‐related pay, position in school league tables and school accountability (Leckie & Goldstein, 2009; Ray *et al*., 2009). Because VA measures compare a student's academic performance to their performance at an earlier stage, they are designed to present a measure of progress in performance that controls for between‐individual time‐invariant differences such as a child's underlying level of ability (McCaffrey *et al*., 2004). They are therefore considered to provide a reliable measure of educational progress independent of the selection of pupils, background characteristics and innate ability (Chetty *et al*., 2014a). This makes them a fairer measure of comparison for the effectiveness of teachers and schools than raw attainment scores, which are confounded by earlier attainment and can unfairly assess the final grades of schools with more disadvantaged student intakes (Leckie & Goldstein, 2009). Contextual value‐added (CVA) measures have also been developed, which additionally account for a range of additional time‐invariant background factors beyond the teacher's or school's control, such as gender, ethnicity, special educational needs and month of birth. There has, however, been debate over which factors should be adjusted for and the extent to which CVA measures adjust sufficiently for these (Todd & Wolpin, 2003). Despite research demonstrating that children assigned to high‐VA teachers outperform children assigned to low‐VA teachers (Chetty *et al*., 2014b), and therefore that VA can correctly identify the most able teachers, there has been criticism of the extent to which they successfully control for time‐invariant factors (Taylor & Nguyen, 2006; Gorard *et al*., 2013).

### What can genetic data offer educational research?

Because VA measures are designed to control for all time‐invariant factors, they should be robust to genetic differences between individuals. This is because the genetic variants that we inherit are fixed at birth and cannot be altered by teacher or school performance. Genetic data therefore offers a unique opportunity to investigate educational performance in novel ways. Over the past decade there has been growing interest in the contribution of genetics towards a range of human behavioural outcomes, including educational attainment. A number of studies using molecular genetics and twin approaches have provided strong evidence that genetics contribute towards educational attainment (Deary *et al*., 2007; Branigan *et al*., 2013; Krapohl *et al*., 2014; Davies *et al*., 2015; Selzam *et al*., 2016). Amongst samples of unrelated individuals, genetic contribution is estimated using a narrow‐sense heritability statistic, which is defined as the proportion of the total variation in attainment that can be explained by genetic variation. For an accessible review of methods for estimating heritability and the drawbacks involved in such methods, see Tenesa and Haley (2013). Heritability is therefore just a correlational statistic and does not imply that a behaviour is immutable, nor does it provide any information on exactly *how* and *why* particular genetic variants associate with outcomes. Furthermore, heritability is dependent upon the trait in question, the population being studied and the spatiotemporal circumstances surrounding the population (Plomin *et al*., 2008; Davey Smith, 2011). Heritability estimates can therefore be expected to vary to a certain degree across studies, but it remains ‘the most useful summary statistic for the genetic contribution to… complex [traits]’ such as education (Tenesa & Haley, 2013, p. 140). Like social inequality, heritability is a useful measure even if the exact mechanisms which generate it are not fully understood. A complete understanding of education is not possible without understanding the contribution of genetics (as well as all other factors), and incorporating genetic evidence could profoundly affect our understanding of education. Including genetic data in studies has the potential to improve estimation of the influence of social factors by reducing residual variation and genetic confounding in statistical models. However, despite increasing sample sizes of genome‐wide association studies (GWASs) used to identify genetic variants that associate with educational attainment, we are still only able to account for a minority of genetic effects. This approach to using data treats genetics not as an unmalleable prognosis of educational attainment, but as a tool to more accurately and reliably assess *modifiable* influences on educational attainment.

A large meta‐analysis of studies estimated the heritability of educational attainment at 40% (95% CI = 35% to 45%) (Branigan *et al*., 2013). There is considerable variation in heritability estimates between twin studies and molecular genetic studies, to an extent due to the former analysing all genetic variance (i.e. including dominance and epistasis), while the latter analyses only additive genetic variance. Using data from the Twins Early Development Study (TEDS), narrow‐sense heritability of attainment at age 16 has been estimated at 31% (95% CI = 7.5% to 54.5%) (Krapohl & Plomin, 2016), half that of the broad‐sense heritability estimate in the same sample of 62% (95% CI = 58% to 67%) (Krapohl *et al*., 2014). While this points to an important contribution of genetics towards educational attainment, it also demonstrates that at least a third of the variation in educational attainment between individuals in TEDS estimated using twin models is due to *non*‐genetic factors. When looking at international or temporal variations in educational outcomes, social factors are likely to be more visible. For example, in the UK, attendance at university has increased hugely over the last couple of generations, far quicker than genetic changes across the population would be possible. Holding time and place constant, genetic variation is likely to explain a greater amount of variation in educational outcomes. This reflects a general principle regarding what generates between‐individual and between‐group differences (Davey Smith, 2011; Keyes & Galea, 2016).

Educational attainment is influenced by many thousands of genetic variants, each of which have a very small effect size rather than a singular (or even small number of) genetic variants which have large effect sizes. The largest GWAS of educational attainment published to date identified only 74 genetic variants with a discovery sample size of 293,723, with the single strongest variant estimated to account for only 0.035% of the variation in educational attainment defined by years of education (Okbay *et al*., 2016). These findings are consistent with a polygenic model in which it is the combination of small effects of many genetic variants that influences a trait.

It is also, of course, important to realise that educational attainment is not a singular construct that can be perfectly measured with a single score. If, in a particular society, use of imagination was valued above focus on strictly following a set curriculum, then what would be considered a good education would differ. There would very likely still be genetic variants that contribute to some children being more imaginative than others, but these would likely not be the same genetic variants that lead some children to more diligently do their homework and revise for exams compared to others.

## Using genetic data to investigate the validity of VA measures

Despite this evidence, educational research has generally been slow to incorporate genetic information (Plomin & Walker, 2003), though some developments have been made (Jerrim *et al*., 2015). Harnessing genetic information offers novel opportunities to investigate educational phenomena. For example, if the goal of VA measures is to control out between‐individual differences and provide a fair assessment of teachers and schools, then they *must* account for genetic factors which directly influence attainment. Recent genetic analyses applied to VA measures have suggested that they are prone to genetic differences and therefore perform poorly at controlling for time‐invariant differences between children. In TEDS, the heritability of a value‐added measure was estimated at 52% (95% CI = 48% to 57%), similar to the heritability of raw educational attainment (Haworth *et al*., 2011). This implies that VA measures do not provide a fair assessment of teachers and schools but are instead genetically biased. The study by Haworth and colleagues used teacher‐reported ability instead of the directly assessed attainment scores which are used to inform school league tables and educational policy. This is important, because teacher‐rated ability is likely to be a less accurate measure of student achievement than National Curriculum test‐assessed point scores. It will be characterised by greater systematic error due to confounding by teacher‐reporting variation on the bias of traits such as ethnicity or physical attractiveness (Burgess & Greaves, 2013; Gershenson *et al*., 2016; Hansen, 2016), and therefore may induce bias in heritability estimates. The heritability for VA measures built from reading and maths test assessments in the same study was similar though, suggesting that such bias may not be present. However, these assessments used were completed online, differed between the two measurement occasions and did not align with the National Curriculum assessments, and so are likely to contain greater measurement error than official data. Further investigation into the ability of VA measures to control for genetic differences between children is required to assess their suitability for informing policy, assessing teacher performance or determining school accountability.

In this study, we exploit molecular genetic data to first estimate the heritability of educational attainment at three time points throughout the compulsory educational lifecourse, and second investigate the ability of VA measures created from examination assessment data to control for time‐invariant between‐individual genetic differences (one of their desirable properties). If VA measures are estimated to have non‐zero heritability, this suggests that they are susceptible to the influence of genetics. Throughout this article, we use the term *heritability* to refer to a measure of heritability calculated from a given set of genetic variants (SNPs, or single nucleotide polymorphisms).

## Methods and materials

### Study sample

Participants were children from the Avon Longitudinal Study of Parents and Children (ALSPAC). Pregnant women were eligible to enrol if they had an expected date of delivery between April 1991 and December 1992 and were resident in the (former) Avon Health Authority area in South West England (for full details of the cohort profile and study design, see Boyd *et al*. (2013) and Fraser *et al*. (2013)). The study website contains details of all the data that are available through a fully searchable data dictionary (ALSPAC data dictionary, available at http://www.bris.ac.uk/alspac/researchers/data-access/data-dictionary/). The ALSPAC cohort is largely representative of the UK population when compared with the 1991 Census data; however, there is under‐representation of some ethnic minorities, single‐parent families and those living in rented accommodation. Ethical approval for the study was obtained from the ALSPAC Ethics and Law Committee and the Local Research Ethics Committees. From the core sample of 14,775 children, 14,115 have data on at least one measure of educational attainment. From these children, genetic data were available for 7,988 after quality control and removal of related individuals. We use the largest available samples in each of our analyses to increase the precision of estimates, regardless of whether a child contributed data to the other analyses.

### Genetic data

In short, DNA of the ALSPAC children was extracted from blood, cell line and mouthwash samples, then genotyped using reference panels and subjected to standard quality control approaches. In full, the children were genotyped using the Illumina HumanHap550 quad chip genotyping platforms by 23andme subcontracting the Wellcome Trust Sanger Institute, Cambridge, UK and the Laboratory Corporation of America, Burlington, NC, USA. The resulting raw genome‐wide data were subjected to standard quality control methods. Individuals were excluded on the basis of gender mismatches; minimal or excessive heterozygosity (where a genetic locus contains two different alleles); disproportionate levels of individual missingness (> 3%) and insufficient sample replication (identity by descent (IBD) < 0.8). Population stratification was assessed by multidimensional scaling analysis and compared with Hapmap II (release 22) European descent (CEU), Han Chinese, Japanese and Yoruba reference populations; all individuals with non‐European ancestry were removed. SNPs with a minor allele frequency of < 1%, a call rate of < 95% or evidence for violations of Hardy–Weinberg equilibrium (HWE) (*P* < 5 × 10^−7^) were removed. Cryptic relatedness (where two individuals in the sample are close relatives, but this is unknown) was measured as proportion of IBD (> 0.1). Related subjects who passed all other quality control thresholds were retained during subsequent phasing and imputation. A total of 9,115 subjects and 500,527 SNPs passed these quality control filters.

Children's genotypes were jointly phased and imputed with the genotypes of the ALSPAC mothers (Illumina human660W quad (mothers)), combining 477,482 SNP genotypes which were in common between the samples. SNPs with genotype missingness above 1% were removed due to poor quality (11,396 SNPs removed) and a further 321 subjects due to potential ID mismatches. This resulted in a dataset of 17,842 subjects containing 6,305 duos and 465,740 SNPs (112 were removed during liftover and 234 were out of HWE after combination). Haplotypes (a group of alleles inherited together) were estimated using ShapeIT (v2.r644), which uses relatedness during phasing. We obtained a phased version of the 1,000 genomes reference panel (Phase 1, Version 3) from the Impute2 reference data repository (phased using ShapeIt v2.r644, haplotype release date December 2013). Imputation of the target data was performed using Impute V2.2.2 against the 1,000 genomes reference panel (Phase 1, Version 3) (all polymorphic SNPs excluding singletons), using all 2,186 reference haplotypes (including non‐Europeans). This gave 8,237 eligible children with available genotype data after exclusion of related subjects using cryptic relatedness measures.

## Education data

### Educational attainment

Our measures of educational attainment are average fine‐graded point scores at each of the major key stages (KS) of education in the UK: KS2 assessed at age 11; KS3 assessed at age 14; and KS4 assessed at age 16. Point scores were used to obtain a richer measure of a child's attainment than level bandings, with the distributions of the raw scores presented in the supporting information Data S1 Section 2. Scores for the ALSPAC cohort were obtained through data linkage to the UK National Pupil Database (NPD), which represents the most accurate record of individual educational attainment available in the UK. We extracted all scores from the KS4 database as this includes attainment at earlier key stages and provides a larger sample size than the earlier databases.

### Value‐added measures

We use two sets of VA measures in our analyses. First, we calculated a raw VA score as the difference between standardised point scores for each student at different key stages. This VA measure can be considered the child's cohort‐specific VA score as it is based upon the rank ordering of the child in the cohort at each occasion. Second, CVA measures were extracted from the NPD linked to ALSPAC participants. The CVA measures—using the example of a CVA score between ages 11 and 14—are calculated as the difference between a child's given exam score (age 14) and the score that would be predicted from that child's previous key stage exam score (age 11). The models used to calculate CVA measures are estimated within a multilevel framework whereby students are nested within schools and the intercept is permitted to vary across schools. The CVA models also account for gender; special educational needs; eligibility for free school meals (a proxy for low income); first language; school mobility; ethnicity; month of birth; an indicator of whether a child has been in care; and residential area level deprivation. We present results for both the raw VA and CVA measures throughout our analyses. Raw and CVA measures are strongly related, with correlations of 0.95 at age 11–14, 0.80 at age 11–16 and 0.78 at age 14–16.

We also use a teacher‐assessed value‐added (TAVA) measure of the ability of children. Teachers are required to grade their students at multiple time points in English, Mathematics and Science on a scale of 1 to 8. These grades reflect the level at which a teacher deems a student to be working, with higher levels reflecting students working at a more advanced stage. Because the levels run throughout a child's educational career, they can be compared at different time points to assess progress. The TAVA measure we use was calculated as the difference between the mean level of English, Mathematics and Science at ages 11 and 14, thus representing progress during these years. Teacher‐reported ability is not available in the NPD at age 16, meaning that our TAVA measure only covers the one educational period. Correlations between the TAVA measure and the VA and CVA measures are 0.333 and 0.405, respectively, far lower than the correlation between the VA and CVA measures of 0.919. We return to this in the discussion.

### Educational attainment polygenic score

An educational attainment polygenic score was generated from genetic data based upon the 74 independent SNPs identified at genome‐wide significance (*p = *5 × 10^−8^) in the largest GWAS of education to date (Okbay *et al*., 2016). Each genetic variant was weighted by the effect size of the variant in the replication cohort of the meta‐analysis, the UK Biobank, and these doses were summed using the allelic scoring function in PLINK (version 1.9) (Purcell *et al*., 2007). The resulting polygenic score provides an estimate of the summed influence that all genetic variants which are identified at GWAS significance have on educational attainment.

### Statistical analysis

To estimate the proportion of variation in educational attainment and VA measures that can be attributed to common genetic variation (SNP heritability), we run a series of univariate analyses using generalised restricted maximum likelihood (GREML) in the software package GCTA. GCTA uses measured SNP level variation to estimate the genetic similarity between every pair of unrelated individuals in the sample and compares this to their phenotypic similarity. Unrelated participants (less related than second cousins) are determined using the ALSPAC Genetic Relatedness Matrices (GRMs). Our univariate analyses are specified as follows:

y=Xβ+g+ϵ

where *y* is the heritability of a phenotype, *X* is a series of covariates, *g* is a normally distributed random effect with variance $\sigma_{g}^{2}$ and *ε* is residual error with variance $\sigma_{\epsilon }^{2}$. Heritability is defined as the proportion of total phenotypic variance (genetic variance plus residual variance) that can be attributed to common genetic variation:

$$h_{\mathrm{SNP}}^{2}=\frac{\sigma_{g}^{2}}{\sigma_{g}^{2}+\sigma_{\epsilon }^{2}}$$

If, across the sample, genetically similar pairs are more phenotypically similar than genetically dissimilar pairs, then heritability estimates of the phenotype will be higher. Population stratification can bias heritability estimates, and can occur if different subpopulations in the sample have systematic differences in allele frequencies due to ancestral differences. To control for these population‐specific variations in allele distributions, the first 20 principal components of inferred population structure are included as covariates in analyses. All continuous variables were inverse normally rank transformed to have a normal distribution, a requirement of GCTA. Power calculations are presented in supporting information Data S1 Section 3, Table S2. Briefly, we are suitably powered to detect heritability estimates greater than 0.15. All code used to generate the results in this study are available from https://github.com/timtmorris/VA-heritability.

## Results

Table 1 displays the number of study children who provide information for each of the analyses and descriptive statistics for each of the variables used. Sample size is higher for the raw attainment measures but reflects some data loss in the raw VA and CVA measures due to missing attainment and background factors, respectively. The CVA measures have higher standard deviations than the raw VA measures because they are measured using differences between predicted point scores and realised point scores, whereas the raw VA measures use differences in standardised score differences.

**Table 1:** Descriptive statistics for children included in the analyses

	*n*	Mean	SD
Age 11 (KS2) points	6,132	28.04	3.85
Age 14 (KS3) points	4,960	35.97	6.19
Age 16 (KS4) points	6,518	39.89	9.48
Age 11–14 (KS2–3) VA score	4,904	0.06	0.42
Age 11–16 (KS2–4) VA score	6,088	−0.03	0.63
Age 14–16 (KS3–4) VA score	4,924	−0.07	0.49
Age 11–14 (KS2–3) CVA score	4,600	0.10	2.52
Age 11–16 (KS2–4) CVA score	6,028	0.98	56.33
Age 14–16 (KS3–4) CVA score	4,914	1.08	45.21

CVA, contextual value‐added; SD, standard deviation; VA, raw value‐added.

### Educational attainment

**Figure 1:**
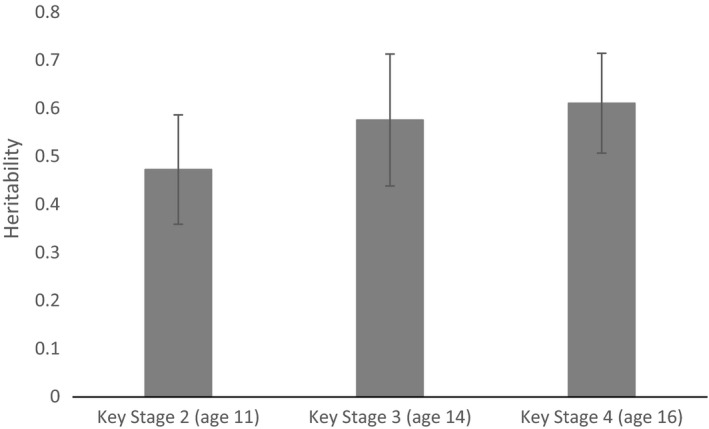
Heritability of key stage average point score attainment. Sample sizes: KS2 = 6,132; KS3 = 4,960; KS4 = 6,518. See supporting information Data S1 Section 4 for full model results.

The SNP heritabilities of educational attainment over time are presented in Figure 1. Heritability rises with age from 47.3% (95% CI = 35.9% to 58.7%) at age 11 to 57.6% (95% CI = 43.9% to 71.3%) at age 14 and 61.1% (95% CI = 50.7% to 71.5%) at age 16. This suggests that in the ALSPAC sample genetic variation contributes towards around half of the total variance in educational attainment using fine‐graded exam scores. These heritabilities are higher than would be expected given the estimated heritability of 40.0% (95% CI = 35.3% to 44.7%) from a meta‐analysis of educational attainment (Branigan *et al*., 2013).

### Value‐added measures

**Figure 2:**
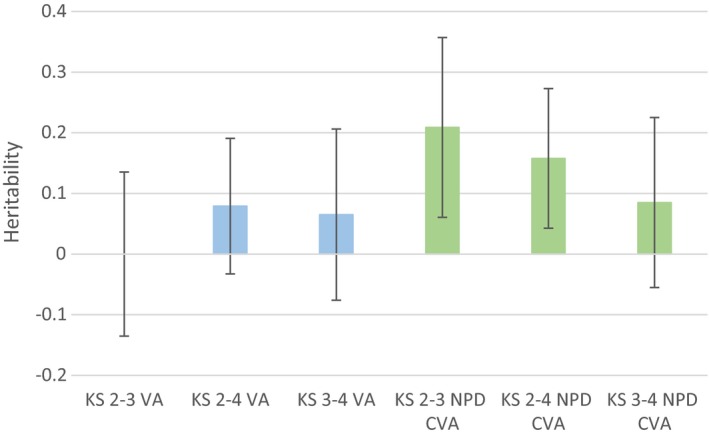
Heritability of value‐added measures. KS, key stage; VA, raw value‐added; CVA, contextual value‐added. Children are aged 11 at KS2, 14 at KS3 and 16 at KS4. Sample sizes: KS2–3 VA = 4,904; KS2–4 VA = 6,088; KS3–4 VA = 4,924; KS2–3 CVA = 4,600; KS2–4 CVA = 6,028; KS3–4 CVA = 4,914. See supporting information Data S1 Section 4 for full model results. [Colour figure can be viewed at http://wileyonlinelibrary.com]

Figure 2 displays the heritability of VA measures, which would be zero if the VA measures control for all time‐invariant genetic differences between children. There is little evidence that common genetic variation can explain the raw VA measures, far smaller than for the raw attainment scores at each key stage. The raw VA measures from ages 11–14, 11–16 and 14–16 show SNP heritabilities of < 0.1% (95% CI = −13.5% to 13.5%), 7.9% (95% CI = −3.3% to 19.0%) and 6.5% (95% CI = −7.6% to 20.6%), respectively. The estimate of heritability for the KS2–3 raw VA score is constrained to zero because the predicted heritability is −0.04, which is outside the bounds of zero to one. The heritability estimates for the CVA measures though are consistently higher than those for the corresponding raw VA measures. There is evidence that the CVA scores are heritable for the period of age 11–14 ($h_{\mathrm{SNP}}^{2}$ = 20.09%; 95% CI = 6.06% to 35.71%) and the period of age 11–16 ($h_{\mathrm{SNP}}^{2}$ = 15.77%; 95% CI = 4.26% to 27.29%). Heritability estimates for the period of age 14–16 are lower, with little strong evidence for a heritable component ($h_{\mathrm{SNP}}^{2}$ = 8.49%; 95% CI = −5.52% to 22.51%). These results consistently suggest that relatively little of the variation in raw VA measures—the unadjusted progress that students make from one key stage to another—can be attributed to common genetic variation. However, they also suggest that the variation in some CVA measures—those that adjust for background factors—*can* in part be attributed to common genetic variation.

To explore this, we ran a series of simulations to determine scenarios in which CVA measures may be more genetically biased than raw VA measures (see supporting information Data S1 Section 1). The simulations demonstrate that CVA measures overstate heritability more than raw VA measures, where the baseline input score contains measurement error (VA $h_{\mathrm{SNP}}^{2}$ = 9.99%; CVA $h_{\mathrm{SNP}}^{2}$ = 16.66% given measurement error of 0.25).

These results contrast with those from a previous study, which estimated heritability of VA measures using teacher‐assessed ability at 50% (Haworth *et al*., 2011). Teacher‐assessed ability is a good proxy for true student ability; the correlations between teacher‐assessed ability and point scores at KS2 and KS3 are 0.884 and 0.921, respectively. However, teacher‐assessed ability may be confounded by teacher‐report bias, which will not be present in directly assessed examination scores. If the baseline measure of achievement is measured with error due to teacher‐reported bias, then it will not fully control for prior achievement. As our simulations demonstrate, this may inflate the estimated heritability. Furthermore, because teacher‐assessed ability is measured using fewer bandings than the rich point scores used by exam assessments, the variability of scores is lower, as children cannot be differentiated within bands. Where the baseline measures are more constrained they will be less effective at controlling for initial differences and may further lead to upward bias in heritability estimates. It is therefore possible that the high heritability observed in the previous study may be due in part to imprecision or teacher bias in the baseline measurement compared to point scores that are traditionally used in educational research.

**Figure 3:**
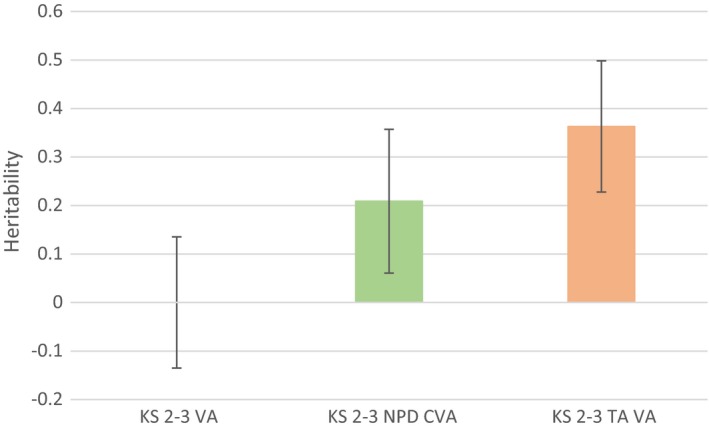
Heritability of KS2–3 value‐added measures. KS, key stage; VA, raw value‐added; CVA, contextual value‐added; TAVA, teacher‐assessed value‐added. Children are aged 11 at KS2, 14 at KS3 and 16 at KS4. Sample sizes: KS2–3 VA = 4,904; KS2–3 CVA = 4,600; KS2–3 TAVA = 5,070. See supporting information Data S1 Section 4 for full model results. [Colour figure can be viewed at http://wileyonlinelibrary.com]

We investigated if the discrepancy between our results and those from the previous study may have reflected genuine differences (a sample issue) or differences caused by alternative methods of assessment (a measurement issue). A TAVA measure similar to that used in the previous study was created for the period of education from age 11 to age 14 (teacher‐rated ability was unavailable at age 16). The ages that the teacher assessments were made were similar to TEDS (ages 10 and 12). Figure 3 presents the GCTA heritability results of our TAVA measure compared to the raw VA and CVA measures at this age. The results suggest a moderate amount of heritability in the TAVA measure of 36.3% (95% CI = 22.8% to 49.8%). This exceeds the heritability point estimates presented in Figures 1 and 2 and suggests that VA measures using teacher‐rated ability are likely to reflect both student progress and genetic differences between students.

### Polygenic score results

**Figure 4:**
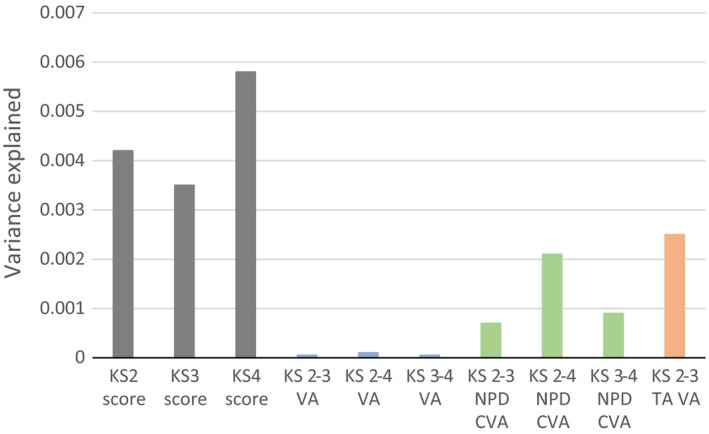
Proportions of variance in outcomes attributed to the EA PGS. KS, key stage; VA, raw value‐added; CVA, contextual value‐added; TAVA, teacher‐assessed value‐added. Children are aged 11 at KS2, 14 at KS3 and 16 at KS4. Sample sizes: KS2 score = 6,132; KS3 score = 4,960; KS4 score = 6,518; KS2–3 VA = 4,904; KS2–4 VA = 6,088; KS3–4 VA = 4,924; KS2–3 CVA = 4,600; KS2–4 CVA = 6,028; KS3–4 CVA = 4,914; KS2–3 TAVA = 5,070. See supporting information Data S1 Section 4 for full model results. [Colour figure can be viewed at http://wileyonlinelibrary.com]

The final stage of the analysis was to investigate the amount of variance in educational attainment and VA measures that an educational attainment polygenic score (EA PGS) predicts. The results (Figure 4) show that the EA PGS accounts for a small but detectable proportion of variance in educational attainment at each key stage, varying from 0.35% to 0.58%. The EA PGS accounts for negligible variation in the raw VA measures (≤ 0.01%), providing further evidence that these successfully account for time‐invariant differences between children and are not influenced by genetic factors. Consistent with the GCTA results, the EA PGS predicts a small but detectable amount of variation in the CVA measures (0.07% to 0.21%), though this is far smaller than the raw attainment scores. Again, this suggests that these adjusted CVA measures are more strongly associated with genetic differences than raw VA measures. The EA PGS explains a greater amount of variation in the TAVA measure (0.25%) than the raw VA (< 0.01%) and CVA (0.07%) measures for this period, closer to the heritability of the age 11 and age 14 point scores themselves. Given that the EA PGS only includes 74 variants, which each have a small effect size, this provides further evidence that value‐added measures based upon teacher‐rated ability are likely to be considerably biased by common genetic variation associated with educational attainment.

## Discussion

### Attainment throughout the educational lifecourse

Our genome‐wide heritability estimates using all common genetic variants provide molecular genetic evidence for a substantial heritable component of educational attainment in ALSPAC, conforming to previous findings from other samples. We find that the heritability of educational attainment increases from 47% at age 11 to 61% at age 16, suggesting that genetics explains a greater proportion of the variation in outcomes at age 16 than earlier ages. This is important, because the age 16 exams play a large role in setting a child's future chances in further education and the labour market. The differences across ages are small and could be due to various factors. For example, parents, teachers and others may work harder at later ages to make the environment more homogenous than at earlier ages. Attitudinal behaviours may also be partly responsible because older children have more freedom to choose their own educational effort with less parental influence. It is also important to note that in some subjects such as Mathematics, children are compelled to enter different tiers based upon ability as assessed after the age 14 exams, which will have placed ceiling and floor caps on the scores that they could attain in the age 16 examinations.

Our estimated heritability is higher than the 40.0% (95% CI = 35.3% to 44.7%) estimated using meta‐analyses (Branigan *et al*., 2013). Furthermore, it is more similar to the broad than narrow‐sense heritability estimated in TEDS = 62% (95% CI = 58% to 67%) and 31% (95% CI = 7.48% to 54.52%), respectively (Krapohl *et al*., 2014; Krapohl & Plomin, 2016). It is possible that this may reflect a true difference whereby the heritability of point scores is higher than that of years of education (as used in the meta‐analysis) or grades (as used in TEDS). However, it must be noted that this may also reflect the fact that the ALSPAC cohort is more spatiotemporally homogenous than other cohorts used in the meta‐analysis; all the children in ALSPAC were born within 3 years in the same geographical location and they mostly experienced the same school system, albeit in a very socially divided, largely urban area. This means there may be a smaller set of environmental factors influencing our results, which could result in reduced environmental variation. This will in turn increase the residual variation in the phenotype that can be attributed to common genetic variation and the resulting heritability estimate.

### Value‐added measures

We found that very little of the variation in raw VA measures built from point score data could be explained by common genetic variation. Surprisingly, we found that these raw VA measures were less heritable than CVA measures, which additionally control for background factors. Our results imply that raw VA measures may be less prone to between‐individual genomic differences than CVA measures and therefore offer a more valid measure for the value added by schools or teachers to a child's education. CVA measures appear to be susceptible to genetic differences between children and may therefore not offer fair assessments of the contribution that teachers and schools make towards a child's educational progress. Our simulations suggest that the reduced performance of CVA measures could be due to measurement error in the baseline scores. Because KS2 and KS3 point scores are determined from a smaller range of subjects than KS4 point scores, they are likely to provide less precise measures of overall academic ability, which results in a baseline measure containing greater measurement error. This measurement error is then inflated when additional contextual factors are accounted for, resulting in CVA measures being more biased by genetic differences than raw VA measures.

It has been argued that adjusting for factors can increase bias where input measures are broad or crude proxies, or related to parental choice (Todd & Wolpin, 2003), and our results provide genetic evidence that supports this. Our simulations may also help to explain why VA measures built from teacher‐reported ability further overestimate heritability. Teacher reports are likely to contain greater measurement error than exam results, and therefore VA measures built from them may demonstrate higher heritability. Careful consideration must therefore be taken when constructing VA measures, and caution should be exercised when using them in educational research or for policy purposes. While the raw measures in our sample appear to be largely independent of genetic background and may provide an *indication* of the contribution that teachers and schools make to a child's educational progress, our findings demonstrate that contextual measures will provide an unfair reflection of teachers and schools and could unfairly penalise those depending upon the intake that they receive. Our results only suggest that CVA measures are less effective at controlling for genetic differences between children than raw VA measures. It is possible, however, that they may be overall less biased and more effective at controlling for other between‐individual social and demographic factors.

The VA measures created from teacher‐rated ability for the same children show considerably higher heritability, of around 36%, suggesting that teacher‐rated ability may be more prone to between‐individual genomic differences than official test point scores data. However, it must be stressed that the error around the point estimates only provides strong evidence for a difference between the raw and teacher‐reported VA measures. Several factors may account for this higher heritability estimate. For example, there is evidence that teacher‐rated ability may be influenced by heritable factors such as attractiveness, which could lead to confounding bias (Clifford & Walster, 1973; Talamas *et al*., 2016). Furthermore, teacher‐rated ability is likely to be a less precise measure of ability than end‐of‐key‐stage point scores, due to its reduced richness as a measure of attainment and therefore reduced imposed variability. As with the CVA measures, if teacher‐rated prior attainment is measured with error or low precision, then heritability estimates may be upwardly biased. A policy implication of this is that baseline measurements need to be accurately measured to eliminate bias, and given that teacher assessments are unlikely to be sufficiently precise, their use as inputs for VA measures should be avoided. The difference in correlations between the TAVA measure and the VA/CVA measures serves to highlight that while teachers are generally adept at assessing their pupils’ overall ability, there is enough error in their assessments that the value‐added measures are biased, leading to a heritable component.

Our teacher‐rated VA estimate contrasts with that from the only previous study examining genetic influences on VA measures, which estimated heritability in TEDS at 50% (Haworth *et al*., 2011). It is possible that this discrepancy arises because the two cohorts represent different samples. However, the two cohorts are drawn from the same country and the age of participants differs only by a maximum of 6 years. It is therefore unlikely that this leads to the observed differences (assuming teachers of the geographically concentrated ALSPAC study were as accurate at determining student ability as the general population of teachers). Second, it is possible that the teacher‐reported ability collected by TEDS may have been measured with greater error, or been subject to greater bias, than that linked from the UK NPD to the ALSPAC cohort. It is likely that a child's level would have been decided with more care in the official (and contractually required) National Curriculum reports used by ALSPAC than the optional survey used by TEDS. Third, the ALSPAC analytical sample is likely to be more genotypically homogenous than the TEDS sample, because it is geographically concentrated rather than national. Fourth, it is also possible that the discrepancy between the TEDS and ALSPAC samples is due to the age at which teacher‐reported ability was measured. ALSPAC measures were taken at ages 11 and 14 (end of KS2 and KS3), while the TEDS measures were taken at ages 10 and 12. It is important to note that in the study by Haworth *et al*. (2011), VA measures built from test assessments had similar heritability to those built from teacher‐rated data, providing a suggestion that teacher‐rated measures may not underperform compared to test data. However, the test data used were not drawn from NPD data but instead from online assessments, used only reading and maths ability, differed between the two measurement occasions and the timing did not align with National Curriculum key stages. This is likely to have resulted in greater measurement error, which our simulations demonstrated could lead to inflation in the resulting VA measures. Furthermore, twin studies may be more susceptible to teacher biases of appearance (Hansen, 2016) than studies of unrelated individuals, because of the similarity of appearance between twins. It is also possible that this discrepancy in findings is at least in part due to differences between GCTA and twin models; however, the discrepancy is likely too large to be fully accounted for by these model differences. Ultimately, further work replicating the GCTA approach is required on other datasets such as TEDS to resolve these differences and determine if they are due to differences in modelling approach or differences in the data measures used.

### Polygenic scores

We found that the EA PGS explained between 0.35% and 0.58% of the variation in educational attainment, depending on the stage of education. The EA PGS explains far less variation in exam scores than the GCTA estimates, because it uses a set of only 74 genetic variants that associate with education at genome‐wide levels of significance. This is a tiny proportion of the genome‐wide variants used by GCTA, resulting in lower explanatory power. This is demonstrated by two recent studies using TEDS data; a genome‐wide score using 108,737 SNPs explained 9% of the variance in age‐16 grades (Selzam *et al*., 2016), whereas a score using only 5,733 SNPs explained 1.5% of the variance in the same trait (Krapohl & Plomin, 2016). While lowering the potential explanatory power of the score, using only the 74 genome‐wide significant variants ensures that our score contains only the variants that are robustly associated with educational attainment. Regarding VA measures, the polygenic score results corroborated with the GCTA results. The EA PGS explained negligible variance in the raw VA measures (≤ 0.01%) but a greater amount of variance in the CVA measures (0.07% to 0.21%) and the teacher‐rated VA measure (0.25%). These results further demonstrate that value‐added measures which adjust for background variables—or those built from teacher‐rated measures of ability—may be confounded by genetics, even when only accounting for 74 variants which associate with educational attainment.

### Limitations

Our results provide only estimates of the variance in educational attainment and value‐added measures across the lifecourse, and do not imply that common genetic variants determine the educational attainment of an individual. The major limitation with this work relates to the potential of GCTA to overestimate heritability, which can occur where model assumptions, particularly that of even linkage disequilibrium between SNPs, are violated (Speed *et al*., 2012). Furthermore, heritability estimates from GCTA are sensitive to the sampling of participants, the accuracy of phenotypic measurement and the structure of the genetic relatedness matrix underlying the data (Krishna Kumar *et al*., 2016). These limitations, however, have been strongly refuted by the authors of GCTA (Yang *et al*., 2016; Yang *et al*., 2017). Nevertheless, our estimates are comparable to those previously conducted using twin designs, despite SNP heritability typically being lower than that derived from twin studies, raising the possibility that our high heritability estimates may suffer from overinflation. One further issue that may lead to overestimation in GCTA heritability estimates is that of dynastic effects, where the parental phenotype/genotype directly affects the offspring's outcomes through the creation of specific types of environment. Such indirect genetic effects, termed ‘genetic nurture’ or ‘environmental bias’, have recently been demonstrated to upwardly bias GCTA heritability estimates of educational attainment, because the methods used here are unable to distinguish between direct and indirect genetic effects (Kong *et al*., 2017; Young *et al*., 2017). Our heritability estimates for educational attainment point scores will also be susceptible to bias by assortative mating, whereby parents non‐randomly select partners based upon level of education, as demonstrated by previous work (Robinson *et al*., 2017). There may also be unobserved differences between individuals (residual population structure) biasing our results; we attempted to account for this by using the first 20 principle components of population structure, however we cannot be certain that these will correct for all differences. This limitation could be overcome with genotypic data on mother–father–offspring trios and future studies should exploit the growing availability of data to investigate this hypothesis. Given that data on teacher‐reported ability was only available for ages 11 and 14, we were unable to examine if the bias in VA measures based upon teacher‐reported ability was consistent between the ages of 11–16 and 14–16. Future studies with teacher‐reported ability at multiple time points should examine any such variation by age. As with all measures based upon educational attainment, random measurement error at the individual level will exist within the data (such as a child suffering illness at the time of examination). However, these random changes will likely provide only a minimal amount of bias at the aggregate level given our sample size. Conversely, teacher‐rated measures could be susceptible to longer‐term factors that may impact a child's educational performance, such as parental illness. It is inevitable that our measure of value‐added will still contain measurement error. Random measurement error will not be related to genetic (or other) underlying factors, and could therefore bias heritability estimates towards the null. However, it is unlikely that our raw VA measure will suffer from heavy bias because the NPD key stage examinations represent the most accurate objective assessment of a child's educational ability. Finally, selection bias may influence our findings due to selective participation in the ALSPAC study (Munafò *et al*., 2017; Taylor *et al*., 2018).

## Concluding remarks

In conclusion, our results demonstrate that common genetic variation contributes towards around half of the total variance in educational attainment measured by exam scores throughout the compulsory educational lifecourse in the ALSPAC sample. Our results also suggest that raw value‐added measures are robust to genomic differences between children but that contextual value‐added measures, which further control for additional background factors and those built from teacher‐reported ability, may be genetically biased. These value‐added measures should therefore be used with caution in educational research and policy, as they have the potential to provide unfair assessment and accountability of teachers or schools, and they may bias performance and position in school league tables.

## Author contributions

TTM, NMD, DD and GDS conceived the study; TTM conducted the analysis; TTM drafted the manuscript. All authors contributed to the final version. TTM will serve as guarantor for the contents of this article.

## Disclosure

The authors declare no conflict of interest.

## Glossary

### Allele

Alleles are the different variant forms of genetic variation found at a specific point on a chromosome. Specific alleles associate with different phenotypic traits (e.g. outcomes such as educational attainment).

### Allele frequency

Allele frequency is the prevalence of a given allele at a genetic locus in the sample, expressed as a decimal, fraction or percentage. The allele frequency is reported in terms of the proportion of alleles that are the effect allele (e.g. the allele associated with higher levels of education) or in terms of the number of minor, or less frequent, alleles (termed *minor allele frequency*).

### Assortative mating

Assortative mating refers to the non‐random manner in which people sort into partnerships with partners who have more similar social and biological characteristics such as height, education and personality than would be expected by chance alone. This may be due to assortment based upon partner choice, convergence in characteristics due to interaction with a partner over time or social homogamy (Robinson *et al*., 2017). Rates of assortative mating may vary between populations and over time.

### Common genetic variation

Common genetic variation refers to all genetic variants across the genome in which the minor or rare allele occurs relatively frequently, that is, above 1%.

### Dominance

Dominance refers to the phenomenon whereby the effect of one allele masks the expression of another allele of a single gene.

### Dynastic effects

Dynastic effects refer to the direct effects of parents’ phenotypes on their offspring. An example of this in the education context would be highly educated parents creating a nourishing learning environment for their children via buying books and helping their children learn to read. These effects are sometimes referred to as ‘genetic nurture’ (Kong *et al*., 2018).

### Epistasis

Epistasis refers to the phenomenon whereby the phenotype of one gene can be modified by others. An epistatic gene refers to the gene whose phenotype is expressed.

### Gene

A stretch of DNA formed by a distinct sequence of nucleotides constituting a section of a chromosome. Coding regions (exons) of the gene encode protein and are interspersed with non‐coding regions (introns). This is distinct from a genetic variant, which can occur in a gene (intragenic) or outside a gene (intergenic).

### Genetic relatedness matrix

Identical twins share all their germline genome and have a genetic relatedness of one. Siblings share half their genetic code and have a genetic relatedness of 0.5. If two unrelated individuals drawn at random from the population are more genetically similar than you would expect by chance, they will have a genetic relatedness of greater than zero. If they are less alike than you would expect by chance, they will have a genetic relatedness of less than zero. The genetic relatedness matrix stores the genetic relatedness between every pair of individuals in a sample. It can be used to estimate heritability using GCTA (see below).

### Genome‐wide association study (GWAS)

A genome‐wide association study (GWAS) tests the associations of hundreds of thousands of genetic variants and an outcome (the phenotype). Due to the number of associations being tested simultaneously, and therefore issues of multiple hypothesis testing, strict *P*‐value thresholds (conventionally 5 × 10^−8^) are used to account for multiple testing. The combination of these strict *P*‐value thresholds and small SNP effect sizes means that GWASs require very large samples. GWASs are typically separated into two parts: an analysis is first performed in a discovery cohort to identify nominally genome‐wide significant SNPs; and second performed in an independent validation cohort to validate these SNPs.

### Genome‐wide complex trait analysis (GCTA)

Genome‐wide complex trait analysis (GCTA) is a statistical programme that uses a genomic‐relatedness‐based generalised restricted maximum likelihood (GREML) approach to estimate the proportion of variance in a phenotype that can be statistically accounted for by all common SNPs. GCTA compares the genetic similarity between unrelated individuals and compares it to their similarity on phenotypic traits; where pairs of unrelated individuals are genetically and phenotypically similar, this provides evidence that phenotypic variation can be explained by genotypic variation. GCTA studies typically require sample sizes in the many thousands.

### Haplotypes

Haplotypes are a specific sequence of alleles that are inherited together from a parent, leading to conserved sequences across generations.

### Hardy–Weinberg equilibrium

Hardy–Weinberg equilibrium refers to the principle that genetic variation across a population will remain constant (in equilibrium) over generations in the absence of external disruptive or evolutionary factors. External factors that may disrupt Hardy–Weinberg equilibrium include non‐random mating, mutations, genetic drift and natural selection.

### Heritability

Heritability is the proportion of total phenotypic variance in a population that can be explained by genetic variance, and therefore ranges from zero (no phenotypic variance explained) to one (all phenotypic variance explained). Broad‐sense heritability (*H* ^2^) is defined as the total proportion of variance in a trait that is explained by all genetic variation, *inclusive* of additive genetic variance, dominance and epistasis (gene–gene interactions). Narrow‐sense heritability (*h* ^2^) is the proportion of total variance in a trait that is explained by additive genetic variance. SNP heritability $h_{SNP^{2}}$ is a measure of narrow‐sense heritability calculated from a given set of genetic variants (SNPs). Heritability is a population rather than an individual parameter, and is specific to both the population and the environment under analysis.

### Heterozygosity

Heterozygosity refers to the occurrence of two *different* alleles at a specific genetic locus.

### Homozygosity

Homozygosity refers to the occurrence of two of the *same* alleles at a specific genetic locus.

### Identity by descent

Where a segment of the genome shared by multiple people is due to inheritance from a common ancestor.

### Linkage disequilibrium

Linkage disequilibrium refers to the combination of alleles at two or more loci occurring more frequently than would be expected by chance. This typically occurs for variants in close proximity in the genome. This is in violation of Mendel's second law of inheritance, which states that the identity of an allele should provide no information about alleles at other points in the genome.

### Phenotype/phenotypic trait

A phenotype is the trait or characteristic of interest, for example: educational attainment, cognition and socioeconomic position are all phenotypes.

### Polygenic trait

Polygenic trait is the term used to refer to phenotypic traits that are influenced by many SNPs, the majority of which can only explain a very small proportion of variance in a trait. Most human behavioural traits that are influenced by our DNA are polygenic, being influenced to a small degree by hundreds or thousands of SNPs.

### Polygenic score

A polygenic score (PGS, sometimes referred to as a *polygenic risk* score) is a summed score of the number of alleles associated with a phenotypic trait. These scores are often weighted by the genetic variant's effect size on the phenotype as estimated from a published GWAS. Polygenic scores can use genetic variants that were associated with the phenotype at different *P*‐value thresholds ranging from genome‐wide significance (*p* < 5 × 10^−8^) to liberal thresholds such as *p* < 0.5. Polygenic scores therefore indicate the summed influence that all genetic variants identified at a given level of GWAS significance have on a phenotypic trait. Because current GWASs are limited in the number of SNPs they can identify, polygenic scores based on these GWAS findings often omit many variants and therefore do not provide an estimate of the total genetic impact on a trait.

### Population stratification

Population stratification occurs when different subpopulations may have systematic differences in allele frequencies due to ancestral differences, such as non‐random mating between subpopulations. These differences can occur because of geographical separation. The association of genetic variants and phenotypes can be confounded by population stratification. To control for population stratification, studies that estimate heritability use principal components analysis (PCA) applied to the genome‐wide SNP data to infer population structure, then include the resultant principal components as covariates in analysis to account for population‐specific variations in allele distributions.

### Single nucleotide polymorphism (SNP)

A single nucleotide polymorphism (SNP) is a genetic variant of a single base pair at a specific position in the genome.

## Supporting information


**Data S1**. Additional information
