# Supplementary material for: Testing the validity of value‐added measures of educational progress with genetic data
Source: Br Educ Res J. 2018 Sep 9;44(5):725–47. doi: 10.1002/berj.3466 (PMC6448053; doi:10.1002/berj.3466)
Supplement: Supplementary file 1 — Data S1. Additional information [file BERJ-44-725-s001.docx]

**Supplementary material to “Testing the validity of value-added measures of educational progress with genetic data”.**

## Section 1: Simulations

To determine scenarios in which contextual value-added measures may suffer from greater genetic bias than raw value-added measures we ran a series of simulations, using the data generating process displayed in Figure S1. Stata code to replicate these simulations is available [here](https://github.com/timtmorris/VA-heritability). A range of scenarios were tested under four categories: equal measurement error on the attainment scores; unequal measurement error on the attainment scores; unequal non-genetically associated covariate effects on the scores; unequal genetically associated covariate effects on the scores.

The results (Figure S2) demonstrate that genetic bias is greater in contextual than raw VA measures where measurement error exists on the input point scores. Furthermore, this phenomenon is purely a function of measurement error on the baseline input score. This represents a problem for VA measures as used in the UK because measurement error is likely to be greater at the earlier KS2 and KS3 timepoints than at the end of compulsory schooling KS4 timepoint. This is due to several factors including for example fewer subjects assessed for the earlier stage assessments and less controlled assessment environments. Genetic bias is greater in raw than contextual VA measures where either non-genetically associated or genetically associated covariates have unequal impacts upon the input point scores.

### Figure S1: DAG of data generating process for simulations. Where “*covar*” represents a non-genetically associated covariate that influences educational attainment and “*gcovar*” represents a genetically associated covariate that influences educational attainment.


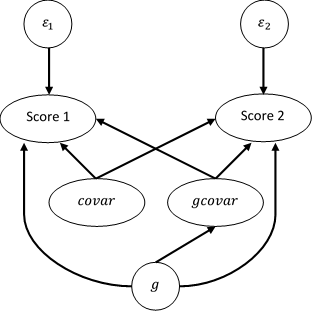


### Figure S2: Simulation results for raw and contextual value-added scores across differing data generating scenarios. CI’s exist on figure but cannot be determined due to precision.

## Section 2: Descriptive information for raw key stage point scores

### Table S1: Descriptive statistics for KS average point scores

|  | n | mean | SD | Minimum | Maximum |
| --- | --- | --- | --- | --- | --- |
| KS2 | 11,108 | 27.46 | 4.06 | 15 | 35.14 |
| KS3 | 9,015 | 34.93 | 6.46 | 15 | 51.54 |
| KS4 | 11,746 | 38.37 | 9.91 | 1.11 | 64.27 |

### Figure S3: Frequency distribution of KS2 average point scores

### Figure S4: Frequency distribution of KS3 average point scores squared

### Figure S5: Frequency distribution of KS4 average point scores squared

## Section 3: Power calculations

We used a GCTA power calculator developed by Visscher et al (2014) which is available at <<http://cnsgenomics.com/shiny/gctaPower/>> to determine the heritability point estimates that we had 80% power to detect given the sample sizes available for each of our analyses.

Visscher et al. (2014) Statistical power to detect genetic (co)variance of complex traits using SNP data in unrelated samples. PLoS Genetics, 10(4): e1004269.

### Table S2: Heritability detectable with 80% power given sample sizes available for analyses

|  | Sample size | Heritability | Power |
| --- | --- | --- | --- |
| KS2 points | 6132 | 0.145 | 0.80 |
| KS3 point | 4960 | 0.180 | 0.80 |
| KS4 points | 6518 | 0.136 | 0.80 |
| KS 2-3 VA | 4904 | 0.181 | 0.80 |
| KS 2-4 VA | 6088 | 0.146 | 0.80 |
| KS 3-4 VA | 4924 | 0.181 | 0.80 |
| KS 2-3 CVA | 4600 | 0.193 | 0.80 |
| KS 2-4 CVA | 6028 | 0.147 | 0.80 |
| KS 3-4 CVA | 4914 | 0.181 | 0.80 |
| KS 2-3 TAVA | 5070 | 0.175 | 0.80 |

## Section 4: Full model results for figures in main manuscript

### Table S2: Full model results from population stratification adjusted univariate analyses of attainment

|  | KS2 points | | KS3 points | | KS4 points | |
| --- | --- | --- | --- | --- | --- | --- |
|  | Estimate | SE | Variance | SE | Variance | SE |
| Genetic variance V(G) | 0.452 | 0.057 | 0.553 | 0.070 | 0.586 | 0.054 |
| Residual variance | 0.504 | 0.054 | 0.407 | 0.067 | 0.373 | 0.050 |
| Phenotypic variance V(P) | 0.956 | 0.017 | 0.960 | 0.020 | 0.959 | 0.017 |
| **Heritability (ratio of V(G) to V(P)** | **0.473** | **0.058** | **0.576** | **0.070** | **0.611** | **0.053** |
| Log Likelihood | -2884.42 |  | -2333.41 |  | -3051.38 |  |
|  | -2920.09 |  | -2368.02 |  | -3122.41 |  |
| Likelihood ratio test | 71.336 |  | 69.226 |  | 142.069 |  |
| p value | <0.001 |  | <0.001 |  | <0.001 |  |
| Sample size | 6132 |  | 4960 |  | 6518 |  |

### Table S3: Full model results from population stratification adjusted univariate analyses of VA measures

|  | KS 2-3 value-added | | KS 2-4 value-added | | KS 3-4 value-added | |
| --- | --- | --- | --- | --- | --- | --- |
|  | Estimate | SE | Variance | SE | Variance | SE |
| Genetic variance V(G) | <0.001 | 0.064 | 0.071 | 0.052 | 0.059 | 0.066 |
| Residual variance | 0.931 | 0.067 | 0.835 | 0.053 | 0.851 | 0.067 |
| Phenotypic variance V(P) | 0.931 | 0.019 | 0.907 | 0.016 | 0.909 | 0.018 |
| **Heritability (ratio of V(G) to V(P)** | **<0.001** | **0.069** | **0.079** | **0.057** | **0.065** | **0.072** |
| Log Likelihood | -2265.67 |  | -2736.71 |  | -2217.51 |  |
|  | -2265.67 |  | -2737.69 |  | -2217.9 |  |
| Likelihood ratio test | 0 |  | 1.979 |  | 0.776 |  |
| p value | 0.500 |  | 0.080 |  | 0.189 |  |
| Sample size | 4904 |  | 6088 |  | 4924 |  |

### Table S4: Full model results from population stratification adjusted univariate analyses of CVA measures

|  | KS 2-3 contextual value-added | | KS 2-4 contextual value-added | | KS 3-4 contextual value-added | |
| --- | --- | --- | --- | --- | --- | --- |
|  | Estimate | SE | Variance | SE | Variance | SE |
| Genetic variance V(G) | 0.200 | 0.073 | 0.143 | 0.053 | 0.075 | 0.063 |
| Residual variance | 0.757 | 0.073 | 0.762 | 0.054 | 0.810 | 0.065 |
| Phenotypic variance V(P) | 0.957 | 0.020 | 0.905 | 0.017 | 0.885 | 0.018 |
| **Heritability (ratio of V(G) to V(P)** | 0.209 | 0.076 | 0.158 | 0.059 | 0.085 | 0.072 |
| Log Likelihood | -2182.178 |  | -2700.32 |  | -2147.2 |  |
|  | -2186.222 |  | -2704.01 |  | -2147.91 |  |
| Likelihood ratio test | 8.087 |  | 7.368 |  | 1.403 |  |
| p value | 0.002 |  | 0.003 |  | 0.118 |  |
| Sample size | 4600 |  | 6028 |  | 4914 |  |

### Table S5: Full model results from population stratification adjusted univariate analyses of teacher assessed VA measures

|  | KS 2-3 teacher assessed value-added | |
| --- | --- | --- |
|  | Estimate | SE |
| Genetic variance V(G) | 0.351 | 0.068 |
| Residual variance | 0.616 | 0.066 |
| Phenotypic variance V(P) | 0.967 | 0.019 |
| **Heritability (ratio of V(G) to V(P)** | 0.363 | 0.069 |
| Log Likelihood | -2425.38 |  |
|  | -2440.34 |  |
| Likelihood ratio test | 29.926 |  |
| p value | 2.24E-08 |  |
| Sample size | 5070 |  |

### Table S6: Variance explained in educational attainment point scores and value-added measures by the educational attainment polygenic risk score

|  | Variance explained | p value | Sample size |
| --- | --- | --- | --- |
| KS2 score | 0.0042 | 2.72 x 10^-07^ | 6,132 |
| KS3 score | 0.0035 | 1.65 x 10^-05^ | 4,960 |
| KS4 score | 0.0058 | 4.02 x 10^-10^ | 6,518 |
|  |  |  |  |
| KS 2-3 VA | <0.0001 | 0.337 | 4,904 |
| KS 2-4 VA | 0.0001 | 0.184 | 6,088 |
| KS 3-4 VA | <0.0001 | 0.539 | 4,924 |
|  |  |  |  |
| KS 2-3 CVA | 0.0007 | 0.038 | 4,600 |
| KS 2-4 CVA | 0.0021 | 2.16 x 10^-04^ | 6,028 |
| KS 3-4 CVA | 0.0009 | 0.019 | 4,914 |
|  |  |  |  |
| KS 2-3 TA VA | 0.0025 | 2.06 x 10^-04^ | 5,070 |

VA, Value-added; CVA, contextual value-added; TAVA, teacher assessed value-added.
